# Effectiveness of a theory-based multicomponent intervention *(Movement Coaching)* on the promotion of total and domain-specific physical activity: a randomised controlled trial in low back pain patients

**DOI:** 10.1186/s12891-017-1788-6

**Published:** 2017-11-06

**Authors:** Andrea Schaller, Katja Petrowski, Timo-Kolja Pfoertner, Ingo Froboese

**Affiliations:** 1 IST-Hochschule University of Applied Sciences, Erkrather Str. 220 a-c, 40233 Düsseldorf, Germany; 2 Institute of Health Promotion and Clinical Movement Science, German Sport University Cologne, Am Sportpark Muengersdorf 6, 50933 Cologne, Germany; 3 Department of Psychology and Psychotherapy, University Witten/Herdecke, Alfred-Herrhausen-Straße 50, 58448 Witten, Germany; 4 Institute for Medical Sociology, Health Services Research, and Rehabilitation Science (IMVR), University of Cologne, 50933 Cologne, Germany; 5 Center for Health through Sport and Movement, German Sport University Cologne, 50933 Cologne, Germany

**Keywords:** Low back pain, Physical activity promotion, Inpatient rehabilitation, Randomized controlled trial

## Abstract

**Background:**

The promotion of physical activity is a major field in rehabilitation and health promotion but evidence is lacking on what method or strategy works best. Ensuing from this research gap, the present study compared the effectiveness of a comprehensive theory based multicomponent intervention *(Movement Coaching)* to a low intensity intervention in low back pain patients.

**Methods:**

A monocenter randomized controlled trial with three measuring points (T0 = baseline, T1 = six month follow-up, T2 = twelve month follow-up) was conducted. *N* = 412 chronic low back pain patients participated. The *Movement Coaching* group (n = 201) received a comprehensive multicomponent intervention with small-group intervention, phone- and web 2.0-intervention. The low intensity control (n = 211) received two oral presentations that were available for download afterwards. Main outcome was total physical activity measured by Global Physical Activity Questionnaire at 12 month follow-up. Additionally, workplace, leisure time and transportation activities were compared. A split-plot anova was conducted for evaluating repeated measure effects and between group effects.

**Results:**

At six and twelve month follow-up there were no statistically significant between group differences in total (T1: p = 0.79; T2: p = 0.30) as well as domain-specific physical activity (workplace (T1: p = 0.16; T2: p = 0.65), leisure time (T1: p = 0.54; T2: p = 0.89), transportation (T1: p = 0.29; T2: p = 0.77) between *Movement Coaching* and the control group. In both groups, workplace physical activity showed the highest proportion of total physical activity. From baseline to twelve month follow-up the results showed a decline in total physical activity (*Movement Coaching:* p = 0.04; control group: p = 0.50).

**Conclusions:**

The comprehensive *Movement Coaching* intervention was not found to be more effective than a low intensity intervention in promoting total and domain-specific physical activity in chronic low back pain patients.

**Trial registration:**

This study is registered at German Clinical Trials Register (DRKS)-ID: DRKS00004878.

## Background

Physical activity is an independent risk factor for non-communicable diseases [1–3]. Population-based surveys assessing physical activity behavior in Germany indicate that only around 25% to 56% of adult men and 16% to 38% of adult women meet the World Health Organization’s recommendations on physical activity [4–6]. Data show that especially people with chronic health conditions are considered to be insufficiently physically active [7, 8]. In consequence, the promotion of physical activity is a major field in rehabilitation and health promotion.

The literature shows that comprehensive and high-quality interventions achieve the most significant long-term increases in physical activity behaviour [9]. The didactics of information delivery should be based on valid behavior change methods (e.g., social cognitive theory and the transtheoretical (or stages of behavior) model [10], setting clear and realistic goals and use simple and specific messages [11]. Though face-to-face interventions are considered the most effective approach [11], long-term interventions such as telephone-based interventions, internet-based interventions and mailed support are considered to increase sustainability [9, 11]. Current research discusses, whether individually tailored interventions are more effective than standard interventions [12, 13].

Assuming that comprehensive, theory based and tailored interventions are more effective at promoting physical activity than standard interventions [9], the aim of the present study was to compare two different interventions: A theory-based multicomponent intervention *(Movement Coaching)* comprising three different components (face-to-face contact, tailored telephone aftercare and internet-based aftercare) and a low-intensity and low cost intervention merely comprising two general presentations on physical activity without theoretical foundation. Owing to the high prevalence of chronic low back pain patients in inpatient orthopaedic rehabilitation in Germany this indication was chosen as a relevant sample for the study.

The research questions assessed were: (1) Is *Movement Coaching* more effective in promoting total physical activity than the low-intensity intervention? (2) Are there differences in leisure time, workplace and/or transportation physical activity between *Movement Coaching* and low-intensity intervention? (3) What are the predictors of an increase in physical activity?

## Methods

### Study design

**Fig. 1:**
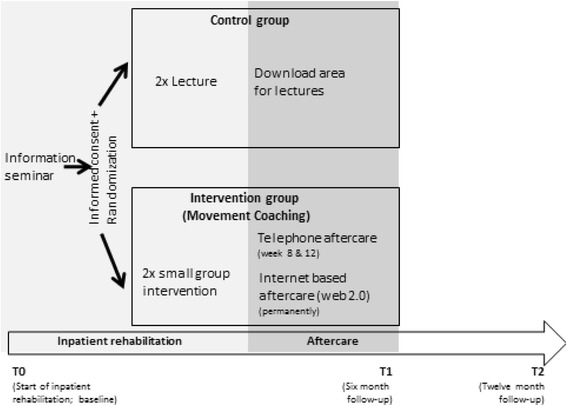
Study design

The study was conducted as a single centre randomised controlled trial with three measuring points (see Fig. 1): T0 = start of inpatient rehabilitation (baseline), T1 = six month follow-up, T2 = twelve month follow-up.

Data were collected by means of a questionnaire on physical activity, sociodemographic and indication-related variables. Following the informational meeting about the study, patients answered the baseline questionnaire at the beginning of inpatient rehabilitation (T0). The outcome data at six months (T1) and twelve months (T2) were collected using a postal questionnaire.

The study was conducted in compliance with the Helsinki Declaration and was approved by the Ethics Committee of the German Sport University Cologne. The study is registered in the German Clinical Trials Register (DRKS00004878). The study protocol for this research project has already been described elsewhere [14].

### Study population

Eligibility criteria included: (1) age 18 to 65 years; (2) starting an inpatient medical rehabilitation treatment due to low back pain. Exclusion criteria included: (1) cognitive impairments; (2) insufficient understanding of the German language; (3) previous surgery within the last three months; (4) posttraumatic conditions (e.g., low back pain following an accident); Written informed consent forms were obtained by all participants. Patients were recruited from May 2013 to April 2014. The twelve months follow-up was completed in April 2015.

As far as possible, the study was conducted single-blinded. Patients were informed about two different physical activity promotion programs. These two programs were labelled *Movement Coaching A* and *Movement Coaching B.* Thus, the patients did not know if they were randomised into the intervention group (*Movement Coaching)* or into the control group (low intensity intervention). The therapist conducted both interventions and was therefore not blinded.

### Intervention

The intervention *(Movement Coaching)* was designed as a multicomponent approach comprising three different components: face-to-face contact (small group intervention, three times during inpatient rehabilitation), tailored telephone aftercare (8 weeks and 12 weeks after rehabilitation) and an internet-based aftercare (web 2.0 platform; available up to six months after rehabilitation).

The theoretical foundation of the multicomponent intervention was the “Rubicon Model of Action Phases” [15]. Additionally, contextual needs were considered within the concept of the intervention [16]. The main objectives and the theoretical foundations of *Movement Coaching* was already published [14].

The face-to-face interventions comprised 60 min each and focused on the formation of intention. The individually tailored telephone aftercare focused on supporting the adoption and maintenance of physical activity in daily routine. Thereby, the contextual needs of the participant (e.g. social acceptance, sociality, look health) were considered. The web 2.0 internet platform obtained further information on health-enhancing physical activity and offered social support by providing a forum to communicate with other participants and the Coach.

Movement Coaching as well as the control intervention were provided by a trained sport scientist with expertise in rehabilitation and health management.

### Control group

The control intervention was designed as a low intensity intervention merely comprising two general presentations on health-enhancing physical activity (30 min each) during inpatient rehabilitation which could be downloaded from a homepage during aftercare.

Differences and similarities of the two intervention strategies as well as the evaluation of the six months follow-up data can be found elsewhere [17].

### Randomization

A randomization schedule was drawn up with a computerized random number generator. To ensure concealment of the treatment allocation, an independent administrative assistant from refonet (rehabilitation research network of the German Pension Fund Rhineland) performed and controlled the randomization.

### Outcome measures

We chose total physical activity (MET-min/week) as our primary outcome as it was the aim of the intervention *Movement Coaching* to promote physical activity in all settings of everyday life (workplace, leisure time, transport).

Physical activity was operationalized by the Global Physical Activity Questionnaire [18, 19], which collects information on workplace physical activity, leisure time physical activity and transport physical activity during a typical week as well as on sedentary time. The Global Physical Activity Questionnaire measures leisure time and workplace physical activities with respect to their intensity. Therefore, the minutes per week for each domain are multiplied by their associated metabolic equivalent (MET)^1^: each minute of vigorous physical activity is multiplied by 8 METs and each minute of moderate physical activity by 4 METs. Transport physical activity is associated with 4 METs per minute. Activity specific scores are summed to give the total MET-min/week [20]. The Global Physical Activity Questionnaire showed that it is valid, reliable and adaptable to incorporate cultural and other differences [21]. Comparison to the International Physical Activity Questionnaire showed moderate concurrent validity (Spearman’s rho 0.45–0.65) and moderate reliability (kappa = 0.67 to 0.73; Spearman’s rho = 0.67 to 0.81) [19]. Compared to objective physical activity (accelerometer data), the Global Physical Activity Questionnaire provided low-to-moderate validity and generally acceptable evidence of reliability [22]. As secondary outcomes, the subscales leisure time (MET-min/week), workplace (MET-min/week) and transportation physical activity (MET-min/week) were assessed.

### Further variables

Anthropometric and sociodemographic variables include *gender, age (years), body mass index (BMI)* and *level of education (“lower secondary school” / “higher level of education than lower secondary school”)*.

Indication-related variables included *duration of Low back pain at the beginning of inpatient rehabilitation (≤12 months / >12 months)* and *pain intensity during the last four weeks* measured by a question from the SF-36 questionnaire (“How much bodily pain have you had during the past 4 weeks?”; answering on a scale from 1 to 6) [23].

### Handling missing data

A base case analysis was performed using data restricted to those patients who replied to the postal six months and the twelve months follow-up questionnaire. In this respect, compliance with the intervention was not considered.

For full case analysis, an intention-to-treat analysis imputing missing data of the outcome variables for the patients that did not reply the questionnaire was performed. Since the effectiveness of physical activity promotion is considered controversial [24–26], we decided to take a conservative approach in the intention-to-treat analysis and imputed based on the principle last observation carried forward (ITT_LOCF_): if six months follow-up data were missing, it was assumed that the data of the outcome variables were the same as at baseline. If twelve months follow-up data were missing and six months follow-up data were available, it was assumed that the data of the outcome variables at twelve months follow-up were the same as at six months follow-up. If six and twelve month follow-up data were missing, it was assumed that the data of the outcome variables at both measuring points were the same as at baseline.

### Sample size calculation

To calculate the sample size, we assumed a difference of 360 MET-min/week between the two groups at six and twelve month follow-up, respectively. This corresponds to 90 min physical activity with moderate intensity which, from the perspective of rehabilitation practitioners, is assumed to be a relevant difference in order to gain a health enhancing effect of physical activity.

We estimated the variance for the sample size calculation from the results of a survey of physical activity in the German population [6]. Sample size was calculated based on a significance level of α = 0.05; power (1-β) = 0.80 and an estimated variance of 1.04. We increased the sample size by 5% because of the high probability for applying a non-parametric test, and we estimated a total of 277 patients for the comparison of two independent groups. By estimating a loss to follow-up of 35% during the twelve months period, we calculated a total sample size of 372 patients, 186 patients per group, respectively.

### Statistical analyses

Means ± standard deviations (SD) and frequency tables (n; %) were calculated to describe the base-case sample at baseline including demographics and anthropometric characteristics. Data were tested on normal distribution by Kolmogorov-Smirnov-Test. Due to the skewed distribution of physical activity data, the median, 25% quartile and 75% quartile were also presented. Beyond, the physical activity data were logarithmized to the base of 10 for further analysis.

Differences in the baseline characteristics between the intervention group *(Movement Coaching)* and the control group (low intensity intervention) were tested using the Mann–Whitney U-test (age, BMI, intensity of pain, leisure time physical activity, workplace physical activity, transport physical activity, total physical activity) and the chi-squared test (gender, education level, duration of low back pain).

For dropout analysis the differences between the patients who replied both, the six months and the twelve months postal follow-up questionnaires, and those patients who did not reply both follow-up questionnaires the sociodemographic and indication-specific variables as well as the baseline variables of the outcome were included as independent variables in the equation of a binary logistic regression model for adjusted evaluation.

For evaluating repeated measure effects and between group effects a split-plot anova was conducted. By this means, the effectiveness of *Movement Coaching* compared to the low intensity intervention was evaluated in regard to total physical activity (question 1) as well as leisure time, transportation and workplace physical activity (question 2). To gain insight in factors associated with an increase of physical activity during the study, we additionally pursued an exploratory evaluation using logistic regression models (question 3). For this purpose we calculated the difference between the 12 month follow up variables and the baseline physical activity variables (T2-T0). Subsequently, we divided into three groups: “increase of physical activity” (T2-T0 > 0), “no change in physical activity” (T2-T1 = 0), “decrease of physical activity” (T2-T0 < 0). All participants showing “no change in physical activity” (T2-T1 = 0) were excluded. In our regression model, the change in physical activity (“increase of physical activity” vs. “decrease of physical activity”) was the dependent variable. We included sociodemographic and indication-related variables as well as baseline physical activity in the model.

For all statistical tests, significance level was set at *p* < 0.05. The Greenhouse-Geisser values were reported in order to correct violation of sphericity in repeated measure (question 1 and question 2). All analyses were run with IBM SPSS Statistics 24.

## Results

Overall, 912 patients were assessed for eligibility, whereof 412 patients (44%) gave informed consent to the study participation and completed the baseline questionnaire. In the patients enrolled for eligibility, there were no differences in sex between participants and non-participants (*p* = 0.18) but the study participants were in average younger than the non-participants (*p* = 0.04). The most frequent reasons mentioned for not participating in the study were concerns in data protection (*n* = 99) and insufficient knowledge of the German language [27].

**Fig. 2:**
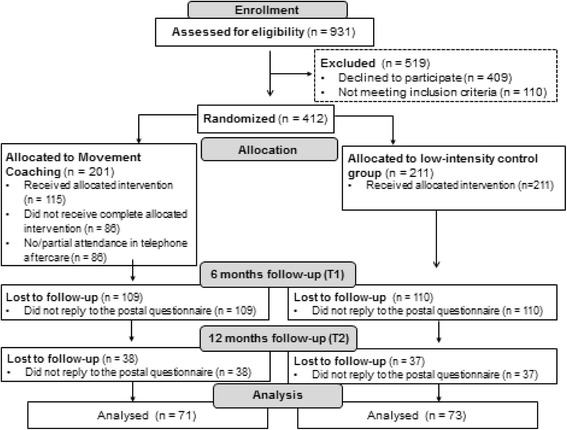
CONSORT Flow-chart

Overall, 35% of the *Movement Coaching* and 35% of the control group completed both, the six months and the twelve months follow-up questionnaire. Therefore, it was possible to analyse 144 questionnaires. Figure 2 shows the CONSORT flow diagram illustrating the progress through the phases of the present study.

### Sample description

A detailed sample description of the full baseline sample already was given in Schaller et al. [17]. Table 1 shows the baseline variables of the present evaluation including the patients that replied to both, the six and twelve month follow-up questionnaire (base case). The base case sample consisted of 97 men and 47 women. The patients in the intervention group *(Movement Coaching)* were significantly younger (*p* = 0.02) and reported more total (p = 0.02) and transportation physical activity (*p* = 0.01) than the patients in the control group.

**Table 1:** Baseline characteristics of the sample that responded both, the six months and twelve months follow-up questionnaires

		Movement Coaching (*n* = 71)	Control group (*n* = 73)	p
Age (years) (*n* = 143)	mean (SD)	50.4 (±7.3)	53.3 (±6.3)	0.02* ^**1**^
Gender: men (n = 144)	n (%)	51 (72%)	46 (63%)	0.23^2^
Body Mass Index (BMI) (*n* = 135)	mean (SD)	28.3 (±4.9)	28.7 (±4.4)	0.46^1^
Highest level of education “lower secondary school” (n = 143)	n (%)	36 (51%)	37 (51%)	0.94^2^
Duration of low back pain: >12 months (*n* = 140)	n (%)	61 (86%)	60 (82%)	0.50^2^
Intensity of pain (minimum = 1; maximum = 6) (n = 135)	mean (SD) median [25%; 75%]-percentile	4.4 (±1.2) 5.0 [4.0; 5.0]	4.4 (±1.0) 4.0 [4.0; 5.0]	0.76^1^
Total physical activity (MET-min/week) (n = 144)	mean (SD) median [25%; 75%]-percentile	8455 (±8664) 5280 [1200; 13,080]	4916 (±6479) 2160 [720; 6480]	0.02* ^**1**^
Workplace physical activity (MET-min/week) (*n* = 144)	mean (SD) median [25%; 75%]-percentile	6228 (±8285) 1440 [0; 1440]	3374 (±5838) 600 [0; 4380]	0.10^1^
Leisure time physical activity (MET-min/week) (n = 144)	mean (SD) median [25%; 75%]-percentile	1187 (±2120) 0 [0; 1200]	863 (±1339) 480 [0; 1020]	0.79^1^
Transport physical activity (MET-min/week) (n = 144)	mean (SD) median [25%; 75%]-percentile	1039 (±1547)400 [0; 1440]	680 (±1949) 0 [0; 440]	0.01* ^**1**^

^1^Mann-Whithney-U-Test; ^2^Pearson-Chi-Quadrat; *significant at significance level *p* < 0.05

### Drop-out analysis

Younger age (*p* < 0.00; OR = 0.95) and lower leisure time activity (*p* = 0.03; OR = 0.85) was significantly associated with a higher chance of not replying to the two postal follow-up questionnaires (see Table 2).

**Table 2:** Factors influencing dropout

*N* = 412	Beta	SE (β)	p	OR
Group “Movement Coaching” vs. “control group”	−0.01	0.24	0.98	0.99
Age (years)	−0.05	0.02	< 0.01*	0.95
Gender: male vs. female	0.13	0.26	0.63	1.14
Body Mass Index (BMI)	0.03	0.02	0.20	1.03
Highest level of education “higher education” vs. “lower secondary school”	−0.32	0.25	0.21	0.73
Duration of low back pain: “>12 months” vs. “≤ 12 months”	0.09	0.34	0.80	1.10
Intensity of pain (minimum = 1; maximum = 6)	0.20	0.13	0.13	1.22
Baseline workplace physical activity (LgMET-min/week)	−0.04	0.06	0.50	0.96
Baseline leisure time physical activity (LgMET-min/week)	−0.17	0.08	0.03*	0.85
Baseline transport physical activity (LgMET-min/week)	−0.05	0.08	0.55	0.96

^1^Adjusted binary logistic regression model (0 = reply; 1 = no reply)

^*^significant at the significance level < 0.05; R^2^ = 0.10

The descriptive baseline variables of the group that replied to both questionnaires (reply) and the group that only replied one or none of the follow-up questionnaires (drop out) are presented in Table 3.

**Table 3:** Drop out analysis: Baseline variables of the patients that replied to the six and twelve month follow-up questionnaire (reply) and dropouts

		Reply (n = 144)	Drop-out (*n* = 268)
Group: Movement Coaching	n (%)	71 (49%)	130 (49%)
Age (years)	mean (SD)	51.9 (±7.0)	49.6 (±8.6)
Gender: men	n (%)	97 (68%)	189 (71%)
Body Mass Index (BMI)	mean (SD)	28.5 (±4.6)	29.8 (±6.0)
Highest level of education: “lower secondary school”	n (%)	73 (51%)	151 (53%)
Duration of low back pain: >12 months	n (%)	121 (84%)	222 (83%)
Intensity of pain (minimum = 1; maximum = 6)	mean (SD) median [25%; 75%]-percentile	4.4 (±1.1) 5.0 [4.0; 5.0]	4.7 (±0.86) 5.0 [4.0; 5.0]
Baseline workplace physical activity (MET-min/week)	mean (SD) median [25%; 75%]-percentile	4781 (±7267) 960 [0; 7720]	6054 (±8292) 780 [0; 10,800]
Baseline leisure time physical activity (MET-min/week)	mean (SD) median [25%; 75%]-percentile	1023 (±1770) 480 [0; 1080]	812 (±1922) 0 [0; 960]
Baseline transport physical activity (MET-min/week)	mean (SD) median [25%; 75%]-percentile	857 (±1766) 0 [0; 945]	699 (±1668) 0 [0; 720]

### Descriptive results and between group effects

For the descriptive results on physical activity at six and twelve month follow-up see Table 4. Neither at six month follow-up nor at twelve month follow-up differences in total physical activity or domain-specific physical activity between *Movement Coaching* and the control were significant (Table 4).

**Table 4:** Descriptive results and between group effects

		Six month follow-up			Twelve month follow-up		
		Movement Coaching	Control group	p^3^	Movement Coaching	Control group	p^3^
**base case** ^**1**^ ** (** ***n*** ** = 144)**		**(n = 71)**	**(n = 73)**		**(n = 71)**	**(n = 73)**	
Workplace physical activity (MET-min/week)	mean (SD) median [25%; 75%]-percentile	1851 (±3922) 660 [0; 2400]	1310 (±3077) 300 [0; 1440]	0.16	1142 (±1576) 375 [0; 1913]	931 (±1256) 405 [0; 1530]	0.65
Leisure time physical activity (MET-min/week)	mean (SD) median [25%; 75%]-percentile	374 (±464) 270 [60; 474]	247 (±286) 180 [83; 310]	0.54	388 (±744) 190 [15; 360]	255 (±306) 180 [45; 360]	0.89
Transportation physical activity (MET-min/week)	mean (SD) median [25%; 75%]-percentile	299 (±557) 120 [0; 315]	265 (±510) 120 [0; 360]	0.29	247 (±485) 88 [0; 338]	224 (±347) 90 [0; 240]	0.77
Total physical activity (MET-min/week)	mean (SD) median [25%; 75%]-percentile	2550 (±4037) 1515 [430; 3005]	1821 (±3158) 800 [250; 2415]	0.79	1679 (±1941) 960 [420; 2805]	1412 (±1301) 960 [390; 2370]	0.30
**ITT**_**LOCF**_^**2**^ **(n = 412)**		**(n = 201)**	**(** ***n*** **= 201)**		**(n = 211)**	**(n = 211)**	
Workplace physical activity (MET-min/week)	mean (SD) median [25%; 75%]-percentile	3791 (±6321) 585 [0; 4455]	4282 (±7615) 360 [0; 3600]	0.58	3303 (±5925) 360 [0; 3510]	3904 (±7218) 450 [0; 3240]	0.94
Leisure time physical activity (MET-min/week)	mean (SD) median [25%; 75%]-percentile	658 (±1799) 180 [0; 540]	514 (±1009) 120 [0; 480]	0.80	641 (±1824) 140 [0; 480]	480 (±918) 120 [0; 480]	0.97
Transportation physical activity (MET-min/week)	mean (SD) median [25%; 75%]-percentile	467 (±1209) 0 [0; 420]	545 (±1439) 60 [0; 420]	0.45	445 (±1194) 0 [0; 420]	531 (±1432) 45 [0; 420]	0.49
Total physical activity (MET-min/week)	mean (SD) median [25%; 75%]-percentile	4867 (±7140) 1830 [350; 5760]	5480 (±8321) 1560 [260; 5940]	0.68	4535 (±6883) 1440 [330; 5155]	4962 (±7908) 1440 [385; 4320]	0.39

^1^patients that replied to the six and twelve months follow-up questionnaire; ^2^ ITT_LOCF_: if six months follow-up data were missing, it was assumed that the physical activity were the same as at baseline, if twelve and six months follow-up data were missing, it was assumed that the physical activity were the same as at baseline, if twelve months follow-up data were missing, it was assumed that the physical activity were the same as at six months follow-up; ^3^ Between group effects of the split-plot anova (LgMET-min/week: the physical activity data were logarithmized to the base of 10 due to skewed distribution), adjustment for multiple comparisons: Bonferroni; ^*^ significant at the significance level < 0.05

**Fig. 3:**
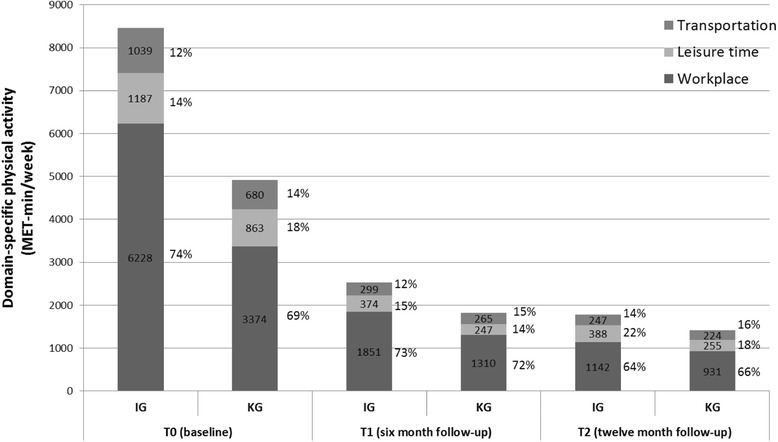
Relative distribution of workplace, leisure time and transportation activities at

Figure 3 presents the distribution of the domain specific physical activities at the three measuring points. In both groups, workplace physical activity showed the highest proportion of total physical activity at each measuring point (see Fig. 3).

**Fig. 4:**
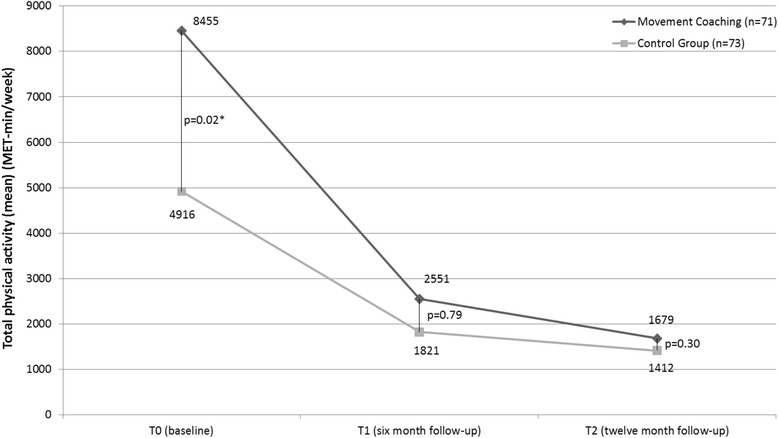
Raw scores of total physical activity at baseline, six month and twelve month follow-up (base case; *n* = 144). Baseline, six and twelve month follow-up; *p*-values: between group differences at T0, T1, T2; ^*^ significant at the significance level < 0.05

The decline in total physical activity did not significantly differ between the intervention and the control group. At six (*p* = 0.79) and twelve month follow-up (0.30) no statistically significant difference between total physical activity in Movement Coaching and the control group could be confirmed (Fig. 4).

### Repeated measure effects

Though group interaction was not statistically significant (*p* = 0.30) the decline in *Movement Coaching* from baseline to twelve month follow-up (*p* = 0.04) was statistically significant, whereas the decline in the control group could not be statistically confirmed (*p* = 0.50). Regarding domain-specific physical activity no statistically significant changes in course of time could be observed. Intention-to-treat analysis confirmed the result of the base case analysis in regard to the primary outcome total physical activity (Table 5).

**Table 5:** Repeated measure effects: within subject significance values and group interaction (base case and intention-to-treat analysis (ITT))

	F	p	Ŋ^2^
Base case^1^ (n = 144)			
Total physical activity			
Time effect	5.69	**0.01** ^*^	0.05
Interaction (Time*Group)	1.17	0.30	0.11
Time effect *Movement Coaching*	3.44	0.04*	0.06
Time effect *control group*	0.70	0.50	0.01
Workplace physical activity			
Time effect	1.38	0.25	0.12
Interaction (Time*Group)	0.45	0.60	0.00
Time effect *Movement Coaching*	1.75	0.18	0.03
Time effect *control group*	0.35	0.71	0.01
Leisure time physical activity			
Time effect	0.81	0.43	0.01
Interaction (Time*Group)	0.54	0.55	0.00
Time effect *Movement Coaching*	1.29	0.28	0.02
Time effect *control group*	0.09	0.91	0.00
Transportation physical activity			
Time effect	0.77	0.46	0.04
Interaction (Time*Group)	4.59	0.01*	0.04
Time effect *Movement Coaching*	1.49	0.23	0.02
Time effect *control group*	3.44	0.04*	0.05
ITT_LOCF_^2^ (n = 412)			
Total physical activity			
Time effect	5.65	0.01*	0.01
Interaction (Time*Group)	2.19	0.13	0.01
Time effect *Movement Coaching*	4.30	0.01*	0.02
Time effect *control group*	0.67	0.51	0.00
Workplace physical activity			
Time effect	4.04	0.03*	0.10
Interaction (Time*Group)	2.12	0.13	0.01
Time effect *Movement Coaching*	4.15	0.02*	0.02
Time effect *control group*	0.32	0.73	0.00
Leisure time physical activity			
Time effect	2.58	0.09	0.01
Interaction (Time*Group)	0.16	0.81	0.00
Time effect *Movement Coaching*	1.40	0.25	0.01
Time effect *control group*	0.58	0.56	0.00
Transportation physical activity			
Time effect	1.51	0.22	0.04
Interaction (Time*Group)	3.19	0.50	0.01
Time effect *Movement Coaching*	0.41	0.67	0.00
Time effect *control group*	3.62	0.03*	0.02

^*^significant at the significance level < 0.05; ^1^Base case analysis: data without imputation of missings; ^2^Intention-to-treat analysis (Last Observation Carried Forward)

### Exploratory subgroup analysis

While 29 (23%) participants showed an increase of total physical activity from baseline to twelve month follow-up (T2), 94 (75%) showed a decrease of physical activity (Table 6). Regarding the domain-specific physical activity levels, again, the number of participants showing a decrease of physical activity in a specific life area was higher. Comparing the domains, leisure time physical activity showed the highest number of participants reporting an increase of physical activity level from baseline to twelve month follow-up (T2) (46 (30%) vs. workplace: 34 (24%), transport: 41 (28%)).

**Table 6:** Change in physical activity: number of participants reporting increase, decrease and no change in physical activity from baseline to twelve month follow-up (T2-T0)

	Increase^1^	No change^2^	Decrease^3^
Total physical activity (n = 144) n (%)	29 (23%)	2 (2%)	94 (75%)
Leisure time physical activity (n = 144) n (%)	46 (30%)	23 (15%)	83 (55%)
Workplace physical activity (n = 144) n (%)	34 (24%)	41 (28%)	69 (48%)
Transport physical activity (n = 144) n (%)	41 (28%)	43 (29%)	65 (44%)

^1^“increase of physical activity” (T2-T0 > 0); ^2^“no change in physical activity” (T2-T1 = 0); ^3^“decrease of physical activity” (T2-T0 < 0)

The sociodemographic and indication-specific variables were included in a regression model (see Table 7). The model on change in total physical activity explained 52% of the variation (R^2^ = 0.52) and the models on domain-specific physical activity explained 70% (workplace physical activity) to 93% (leisure time physical activity). In all models, baseline physical activity was statistically significantly associated with change in physical activity. None of the sociodemographic and indication-related variables included in the regression model showed an association with change in total physical activity. Regarding domain-specific physical activity, results showed one single statistically significant association: a higher body mass index was associated positively with an increase in transport physical activity (OR = 1.304; 95% confidence interval: [1.049; 1.620]; *p* = 0.017).

**Table 7:** Associated factors with an increase of physical activity

N = 144	Beta	SE (β)	Sig.	OR	95%-CI
Total physical activity^1^					
Group: Control group vs. Movement Coaching	−0.024	0.792	0.976	0.976	[0.207; 4.608]
Age (years)	0.027	0.064	0.668	1.028	[0.907; 1.164]
Gender: men vs. women	0.739	1–005	0.462	2.094	[0.292; 15.023]
Body Mass Index (BMI)	0.027	0.102	0.789	2.094	[0.841; 1.255]
Highest level of education: “higher than lower secondary school” vs. “lower secondary school”	−0.364	0.837	0.663	0.695	[0.135; 3.582]
Duration of LBP at baseline: “>12 months” vs. “≤12 months” vs.	−0.978	1.063	0.358	0–376	[0.047; 3.025]
Intensity of pain at baseline(minimum = 1; maximum = 6)	−0.162	0.424	0.702	0.850	[0.371; 1.950]
Baseline physical activity (MET-min/week)	−0.001	0.001	0.018*	0.999	[0.998; 1.000]
Leisure time physical activity^2^					
Group: Control group vs. Movement Coaching	−2.749	2.088	0.188	0.064	[0.001; 3.833]
Age (years)	0.157	0.219	0.473	1.170	[0.762; 1.797]
Gender: men vs. women	1.425	1.819	0.433	4.158	[0.118; 146.877]
Body Mass Index (BMI)	0.252	0.176	0.153	1.287	[0.911; 1.818]
Highest level of education: “higher than lower secondary school” vs. “lower secondary school”	1.877	1.831	0.305	6.536	[0.180; 236.743]
Duration of LBP at baseline: “>12 months” vs. “≤12 months” vs.	−0.135	2.285	0.953	0.873	[0.010; 76.946]
Intensity of pain at baseline(minimum = 1; maximum = 6)	0.419	0.608	0.491	1.520	[0.461; 5.010]
Baseline physical activity (MET-min/week)	−0.021	0.008	0.005*	0.979	[0.965; 0.994]
Workplace physical activity^3^					
Group: Control group vs. Movement Coaching	−0.539	0.859	0.530	0.583	[0.108; 3.141]
Age (years)	0.081	0.068	0.230	1.085	[0.950; 1.239]
Gender: men vs. women	0.873	0.854	0.307	2.395	[0.449; 12.774]
Body Mass Index (BMI)	0.038	0.106	0.721	1.039	[0.844; 1.278]
Highest level of education: “higher than lower secondary school” vs. “lower secondary school”	0.928	0.895	0.300	2.531	[0.438; 14.629]
Duration of LBP at baseline: “>12 months” vs. “≤12 months” vs.	−1.960	1.609	0.223	0.141	[0.006; 3.298]
Intensity of pain at baseline(minimum = 1; maximum = 6)	−0.208	0.405	0.607	0.812	[0.367; 1.769]
Baseline physical activity (MET-min/week)	−0.002	0.004	0.001*	0.998	[0.997; 0.999]
Transport physical activity^4^					
Group: Control group vs. Movement Coaching	−0.770	0.948	0.417	0.463	[0.072; 2.968]
Age (years)	0.078	0.071	0.269	1.081	[0.941; 1.241]
Gender: men vs. women	0.471	0.919	0.608	1.601	[0.264; 9.707]
Body Mass Index (BMI)	0.265	0.111	0.017*	1.304	[1.049; 1.620]
Highest level of education: “higher than lower secondary school” vs. “lower secondary school”	0.533	0.856	0.538	1.703	[0.313; 9.279]
Duration of LBP at baseline: “>12 months” vs. “≤12 months” vs.	−1.242	1.617	0.442	0.289	[0.012; 6.870]
Intensity of pain at baseline(minimum = 1; maximum = 6)	0.878	0.543	0.106	2.405	[0.830; 6.970]
Baseline physical activity (MET-min/week)	−0.006	0.002	<0.000*	0.994	[0.991; 0.997]

Dependent variable: change in physical activity from baseline to twelve month follow-up (T2-T0) (“increase of physical activity”; T2-T0 > 0) vs. “decrease of physical activity”: T2-T0 < 0); ^1^R^2^ = 0.52; ^2^R^2^ = 0.93; ^3^R^2^ = 0.70; ^4^R^2^ = 0.77; *p < 0.05

## Discussion

The results of the present study showed that *Movement Coaching* was not more effective in promoting total physical activity and domain-specific physical activity compared to a low-intensity control intervention (question 1 and question 2). A noticeable aspect of the present study is that, in both groups, total physical activity declined significantly during the twelve months follow-up period. Subgroup analyses on predictors of an increase of physical activity from baseline to twelve month follow-up predominantly identified a negative association between a higher baseline physical activity and a decrease in physical activity during the twelve month period. Besides, a higher body mass index was associated positively with an increase in transport physical activity (question 3).

Even though the results of the present study cannot contribute evidence on a superior strategy in physical activity promotion it provides novelty to the field of physical activity promotion.

The consideration of physical activity in different areas of life (workplace, leisure time, transportation) provides important implications for the future field of practice concerning physical activity promotion. In retrospect, for example, the comparatively low level of education in our sample would have needed further consideration for tailoring the intervention. As a low level of education tends to be associated with higher workplace activity [28] as well as a lower physical activity during leisure time [28–30] the question arises, whether interventions promoting physical activity targeting persons with low level of education should rather focus on leisure time physical activity instead of total physical activity. The World Health Organization guidelines recommend at least 150 min of moderate-intensity or 75 min of vigorous-intensity aerobic physical activity throughout the week, what corresponds to 600 MET-min/week. The recommendations relate not only to sports, but also include explicitly other leisure time activities, workplace and transport activity [31]. Even though more than 50 % of the sample achieve the recommendations based on total physical activity (see Table 4), workplace physical activity contributed the largest share of physical activity performed. In contrast, the results on leisure time physical activity (see Table 4) indicated that less than 25% of the sample were engaged in leisure time physical activity during leisure time that was consistent with the World Health Organization’s recommendations to achieve health benefits. Ensuing from the highly different proportions of workplace and leisure time physical activity in total activity, a discussion about the same effects of workplace, leisure time and transportation physical activity on health should be started. Therefore, further studies on the association of physical activity in different areas of life and health status are of utmost importance.

The decline in physical activity in both groups is astonishing at first glance and seems to contradict current research showing moderate evidence for the increase of physical activity through different interventions [9, 25]. A possible explanation for the decline in physical activity might be the difficulties in measuring physical activity by a questionnaire. Literature shows a low correlation of objectively and subjectively measured physical activity [32, 33]. More particularly, a study from van Weering et al. (2011) showed that low back pain patients appear to have even more problems in estimating their physical activity levels than healthy people [34]. The assumption of an unrealistic self-assessment is supported by an additional study with the same sample comparing subjective and objective physical activity. Thereby, no significant correlations between subjective and objective results could be proved and self-reported data showed an overestimation of 46 min/day (vigorous intensity) to 78 min/day (moderate intensity) [35]. The second possible explanation could be the setting of the intervention, an inpatient rehabilitation center. As the facilitation of self-competence is an integral component of rehabilitation, it is reasonable to assume that patients might have improved their self-assessment during the intervention period. By implication, they probably overestimated their physical activity levels at the beginning of inpatient rehabilitation even more.

The results of our exploratory analyses might underline this assumption as we showed that higher baseline physical activity levels were associated with a decrease in physical activity during the twelve month period. Beyond, several studies supported the assumption that the overestimation of physical activity might be associated to a higher body mass index [36–38] what might be supported by the association of body mass index and the increase of transport physical activity shown in our results. Hence, regarding the practical implications it needs to be considered, that our additional exploratory evaluation only gives information on factors associated with an increase in physical activity: no information on the amount of increase and its relevance on health can be given.

Overall, our study includes a number of important strengths. One such strength was the domain-specific measurement of physical activity. Besides this, choosing a low intensity intervention as a control group instead of a non-intervention control group was undoubtedly a strength of the present study. Since it was already assumed that the promotion of physical activity exerts an influence, the study went further and compared different approaches. Based on this, discussions on resource allocation in physical activity promotion might be opened. Yet, another methodological strength was the randomized controlled study design providing high internal validity of results and mostly regarded as the “golden standard” for evaluation in health care.

Nevertheless, the present study has several limitations. Certainly, the main limitation of the present study is the high drop-out. This has several consequences regarding the interpretation of the results. First, the findings should be interpreted with caution due to the fact that we did not achieve the calculated sample size and therefore the study is underpowered to detect the assumed between group differences at six and twelve month follow-up, respectively. Second, due to the associated problem of imputing missing values, especially the results regarding the decline of physical activity during the course of the study should be interpreted very cautiously. Third, a potential bias resulting from the non-reply to the postal six and twelve month follow-up questionnaires needs to be taken into account. We were not successful to increase the response rate during the conduction of the study, eeven though we integrated a repeated sending of the questionnaire if the participant did not reply within two weeks as well as incentives (all participants who sent back the postal six/twelve month follow-up questionnaires went into a draw to win a tablet computer and a voucher for a wellness-weekend). A further limitation is, although, the randomised controlled design assures a high internal validity this study design, that it is exposed to several problems in rehabilitation practice, like the patients recognising that they were receiving different types of treatment. Therefore, the authors cannot completely exclude the possibility of bias resulting from the exchange of information between the patients. To substantiate the robustness of the results missing values were imputed conservatively and an intention-to-treat analysis with last observation carried forward was calculated. This confirmed the robustness of the results in regard to the primary outcome total physical activity. However, the dropout rate in the present study is comparable to other studies evaluating multicomponent lifestyle interventions [39, 40]. A third limitation is the use of a self-reported physical activity measurement. Besides a potential bias due to social desirable answers and recall bias, validity of physical activity questionnaires needs to be questioned [33]. However, self-administered physical activity questionnaires are the most commonly used as this is the most inexpensive method to use in large-scale studies [41] and no differences in measurement or recall bias between the two groups in the present study were expected.

## Conclusion

Our study brings up the important question if less is more in regard to physical activity promotion as our results could not statistically confirm the superiority of the comprehensive multicomponent intervention (Movement Coaching) compared to the low intensity control intervention. On the one hand, this contradicts the assumption that comprehensive and high-quality interventions achieve the most significant long-term increases in physical behaviour [9]. On the other hand, other studies comparing one intervention to another intervention instead of a non-intervention control group could not proof the superiority of a specific intervention strategy in physical activity promotion neither [12, 13, 42–45]. As the importance of physical activity promotion in prevention and rehabilitation is beyond discussion further research not only on the effectiveness but also cost-effectiveness of different interventions promoting physical activity is needed.

## Abbreviations

BMI: Body Mass Index
e.g.: exempli gratie/for example
Lg: logarithm
MET: metabolic equivalent
**min**: minutes
SD: standard deviations
T0: baseline
T1: six month follow-up
T2: twelve month follow-up

## Footnotes

1 The metabolic equivalent (MET) is a physiological measure expressing the expended energy of physical activities. MET is defined as the ratio of the rate of energy consumption during a specific physical activity to a reference metabolic rate.
